# Supplementary material for: Identification of photoperiod-induced specific miRNAs in the adrenal glands of Sunite sheep (Ovis aries)
Source: Front Vet Sci. 2022 Jul 22;9:888207. doi: 10.3389/fvets.2022.888207 (PMC9354845; doi:10.3389/fvets.2022.888207)
Supplement: Supplementary file 1 [file Data_Sheet_1.ZIP › Supplementary file/miRDeep2 file.zip!/miRDeep2 file/Know_Structure/SPLP42A1/oar-mir-30b.pdf]

miRBase precursor : oar-mir-30b
Total read count : 24416
oar-miR-30b read count : 24218
remaining reads : 198

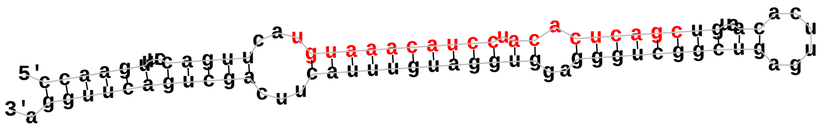

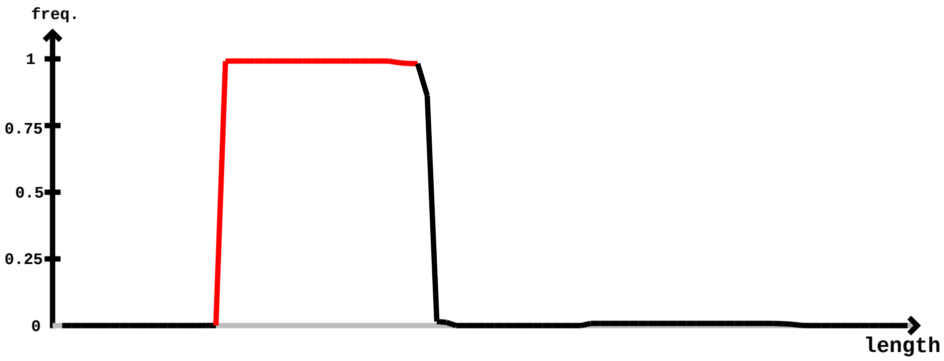

oar-miR-30b

```
5'- ccaaguuuccaguucauguaaacauccuacacucagcuguaacacuugagucggcugggagguggauguuuacuucagcugacuugga   -3'    exp
    (((((...(((((...(((((((((((.((.((((((((..((......))))))))))..))))))))))))...))))))))))).    reads      mm         sample
    ..............cauguaaacauccuacacucagcu..................................................    2          0          seq
    ...............auguaaacauccuacacucagcu..................................................    1          0          seq
    ...............auguaaacauccuacacucagcugu................................................    1          0          seq
    ................uguaaacauccuacacu.......................................................    3          0          seq
    ................uguCaacauccuacacu.......................................................    1          1          seq
    ................uguaaacauccuacacuU......................................................    39         1          seq
    ................uguaaacauccuacacuc......................................................    103        0          seq
    ................uguaaacauccuacacuca.....................................................    75         0          seq
    ................uguaaacauccuacacucag....................................................    17         0          seq
    ................uguaaacauccuacacucagc...................................................    2929       0          seq
    ................uguaaacauccuacacucagcu..................................................    20671      0          seq
    ................uguaaacauccuacacucagcug.................................................    75         0          seq
    ................uguaaacauccuacacucagcugu................................................    291        0          seq
    ................uguaaacauccuacacucagcugua...............................................    1          0          seq
    .................guaaacauccuacacucagc...................................................    1          0          seq
    .................guaaacauccuacacucagcu..................................................    8          0          seq
    ......................................................cugggagguggauguuuac...............    2          0          seq
    ......................................................cugggagguggauguuuacu..............    25         0          seq
    ......................................................cugggagguggauguuuacuu.............    56         0          seq
    ......................................................cugggagguggauguuuacuuc............    102        0          seq
    ......................................................cugggagguggauguuuacuuca...........    10         0          seq
    .......................................................ugggagguggauguuuacu..............    2          0          seq
    .......................................................ugggagguggauguuuacuu.............    1          0          seq
```
